# Implicit learning of artificial grammatical structures after inferior frontal cortex lesions

**DOI:** 10.1371/journal.pone.0222385

**Published:** 2019-09-20

**Authors:** Tatiana Jarret, Anika Stockert, Sonja A. Kotz, Barbara Tillmann

**Affiliations:** 1 CNRS, UMR5292, INSERM, U1028, Lyon Neuroscience Research Center, Auditory Cognition and Psychoacoustics Team, Lyon, France; 2 University Lyon 1, Villeurbanne, France; 3 Language and Aphasia Laboratory, Department of Neurology, University of Leipzig, Leipzig, Germany; 4 Dept. of Neuropsychology, Max Planck Institute for Human Cognitive and Brain Sciences, Leipzig, Germany; 5 Faculty of Psychology and Neuroscience, Dept. of Neuropsychology, Maastricht University, Maastricht, The Netherlands; 6 Faculty of Psychology and Neuroscience, Dept. of Psychopharmacology, Maastricht University, Maastricht, The Netherlands; Universidad de Salamanca, SPAIN

## Abstract

**Objective:**

Previous research associated the left inferior frontal cortex with implicit structure learning. The present study tested patients with lesions encompassing the left inferior frontal gyrus (LIFG; including Brodmann areas 44 and 45) to further investigate this cognitive function, notably by using non-verbal material, implicit investigation methods, and by enhancing potential remaining function via dynamic attending. Patients and healthy matched controls were exposed to an artificial pitch grammar in an implicit learning paradigm to circumvent the potential influence of impaired language processing.

**Methods:**

Patients and healthy controls listened to pitch sequences generated within a finite-state grammar (exposure phase) and then performed a categorization task on new pitch sequences (test phase). Participants were not informed about the underlying grammar in either the exposure phase or the test phase. Furthermore, the pitch structures were presented in a highly regular temporal context as the beneficial impact of temporal regularity (e.g. meter) in learning and perception has been previously reported. Based on the Dynamic Attending Theory (DAT), we hypothesized that a temporally regular context helps developing temporal expectations that, in turn, facilitate event perception, and thus benefit artificial grammar learning.

**Results:**

Electroencephalography results suggest preserved artificial grammar learning of pitch structures in patients and healthy controls. For both groups, analyses of event-related potentials revealed a larger early negativity (100–200 msec post-stimulus onset) in response to ungrammatical than grammatical pitch sequence events.

**Conclusions:**

These findings suggest that (i) the LIFG does not play an exclusive role in the implicit learning of artificial pitch grammars, and (ii) the use of non-verbal material and an implicit task reveals cognitive capacities that remain intact despite lesions to the LIFG. These results provide grounds for training and rehabilitation, that is, learning of non-verbal grammars that may impact the relearning of verbal grammars.

## Introduction

The left inferior frontal gyrus (LIFG) and in particular BA 44/45 (i.e. Broca’s area) has been associated with the processing of structure in various domains, such as syntactic structure in language [1,2], syntactic-like structure in music [3] as well as the acquisition and processing of artificial grammars or new language systems [4,5]. These neuroimaging data are consistent with brain stimulation data [5] and with lesion evidence in this region [6,7]. For example, patients with LIFG lesions display deficits in syntax processing [8,9].

The use of artificial grammars has extended our understanding of the LIFG’s role in the acquisition of new syntactic structures and, once knowledge is acquired, in the processing of syntactic structures as well as in the response to violations of syntactic structures. For example, the use of artificial grammar and artificial language allows manipulating various features of the to-be-acquired structures, such as local and hierarchical, long-distance structures [10]. This allows for a more detailed view on the role of Broca’s area in language processing [11]. More generally, it has been suggested that studying implicit learning (even when using non-verbal materials) allows investigating cognitive sequencing in general [12].

Research investigating the acquisition of artificial grammar and artificial language has utilized both explicit and implicit learning paradigms. In an explicit paradigm, participants are instructed to learn and/or extract underlying grammatical rules [13]. In an implicit paradigm, participants are exposed to an artificial grammar/language system without being informed about it [4] or without being told about the underlying rules [14]. The implicit paradigm permits to exploit implicit cognitive abilities similar to the process of learning a first language or getting enculturated to the musical system of one’s culture [15], which often happens via mere exposure and has been shown to be more powerful than explicit approaches, in particular for patients or the elderly [16].

**Fig 1:**
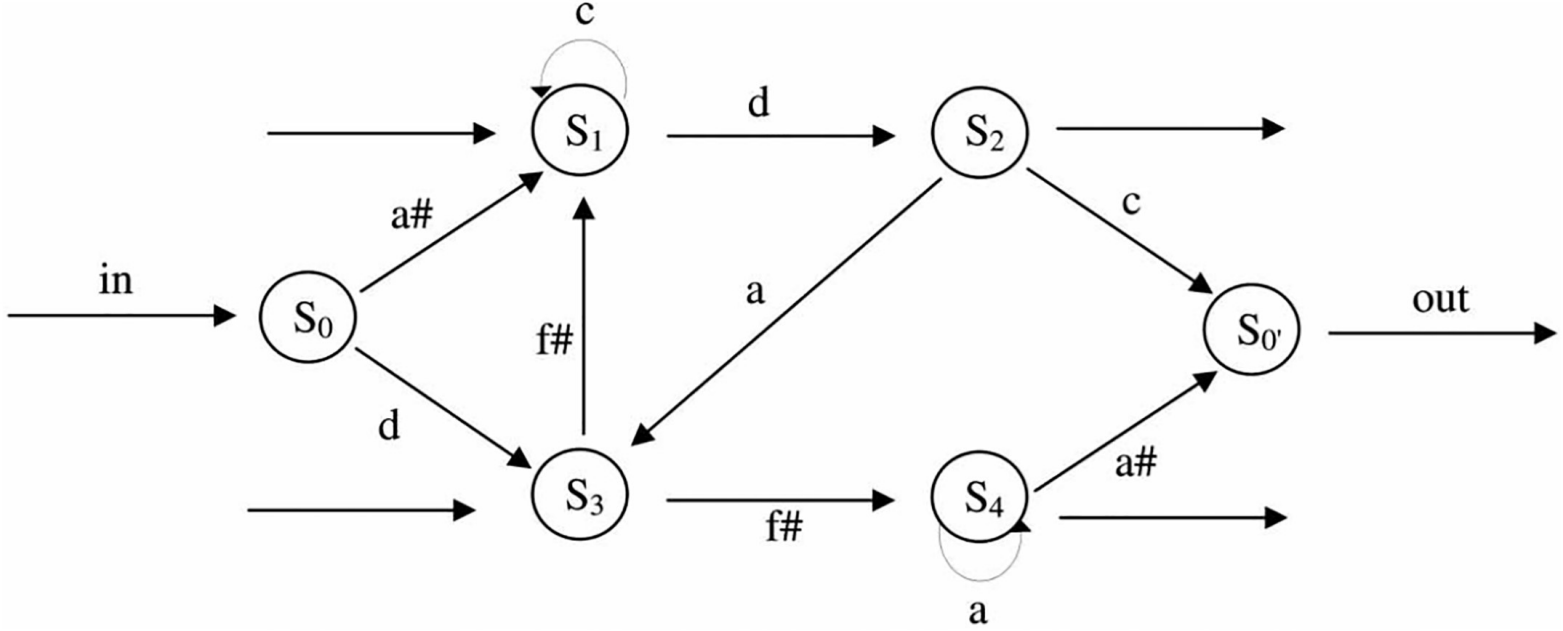
Finite-state grammar used for the construction of the tone sequences. Adapted from Tillman, B. and Poulin-Charronnat, B. Auditory expectations for newly acquired structures. *Quarterly Journal of Experimental Psychology* 63(8), pp. 1646–1664. Copyright 2010 by The Experimental Psychology Society. Reprinted by permission of SAGE Publications, Ltd.

The investigation of incidental or implicit learning of a new structural grammar was first introduced by Reber (1967) using an artificial grammar learning paradigm [17]. A typical experiment contains two phases: In the first phase (exposure phase), participants are exposed to grammatical sequences created from a finite-state grammar based on a set of events (e.g., written letters). Fig 1 gives an example of a finite-state grammar (used in the present study) that visualizes the set of rules determining the structure of the sequences by following the arrows (i.e., valid transitions) and chaining elements together in a sequence. In a second phase (test phase), participants are informed that all sequences of the exposure phase are created according to a set of grammatical rules and are then asked to perform a classification task on a set of new sequences that either meet the rules of the newly acquired grammar or violate these rules. Generally, participants perform above chance in the test phase, even though they often cannot explain their choice. This suggests implicit learning of artificial grammar structures [17]. The grammar learning paradigm has also been used with non-verbal material such as musical timbre [18] or tones differing in pitch [19,20]. Dependent on the types of new sequences used in the test phase, one can conclude for the acquisition of more or less sophisticated knowledge by the participants. For example, introducing new elements or chunks in the ungrammatical items represent mere local violations and do not allow conclusions in how far knowledge about grammatical structures was acquired or not [21–23].

After exposure to an artificial grammar, electrophysiological correlates of artificial grammar violations include the N1, N2 and N2/P3 components of the event-related potentials (ERPs) [24–26]. For example, the N2/P3 complex appears when a violation of acquired explicit knowledge occurs, while an N2 response to ill-formed sequences occurs after implicit knowledge acquisition [27,28]. Neuroimaging studies have reported the involvement of the LIFG in artificial grammar learning [29,30] as well as the acquisition of natural language syntax in the laboratory [14]. For example, after exposure to visually presented sequences of consonants, created in an artificial grammar, Broca’s area was activated during the processing of well-formed artificial sequences (respecting the grammar). This activation was increased for sequences that violated the artificial grammar [30]. Other studies reported activation of the LIFG and its right-hemisphere homologue (even though to a lesser extent) in artificial grammar learning based on verbal materials [14,31,32].

Patient evidence on the learning and processing of artificial grammars is controversial [33–35]. For example, Broca’s aphasics and agrammatic aphasics had difficulties in the implicit learning of a grammar of auditorily presented letters [33] and visually presented shapes [34], while other agrammatic patients were able to learn structures implemented with simple visual word associations in pictures [35]. One reason for unimpaired structure learning may be the fact that some studies used relatively strong violations of the learned grammar in the test phase, restricting however, solid conclusions for structure learning [21,23]. These violations might include new, previously not encountered event combinations, leading to an alternative hypothesis for observed data patterns, which are not reflecting learning of grammatical features, but rather the detection of new bigrams or unseen repetitions [22]. Furthermore, some of these studies relied on variant serial reaction time paradigms that test one repeatedly presented sequence rather than various structures based on a set of new artificial grammatical rules.

Another reason for discrepant results may come from diverse lesion sites and types. For example, the patients in Christiansen et al. (2010), showing artificial grammar learning deficits, were rather heterogeneous with respect to lesion sites (Broca’s area or extended fronto-temporal regions) [34]. It is also worth noting that lesions in aphasic patients may extend to subcortical regions such as the basal ganglia (BG) [36,37]. The BG are involved not only in movement control but also play a role in higher cognitive functions, such as learning, sequencing, and temporal processing [38]. Consequently, patients with extended and/or ill-defined, heterogeneous lesion sites do not allow investigating the critical role of the LIFG in a network supporting the learning and processing of grammatical structures.

The present study tested the implicit learning of an artificial pitch grammar in patients with well-described lesions in the vicinity of the LIFG excluding subcortical lesions. We aimed at investigating whether the LIFG impacts structure learning as suggested by previous research. We used an artificial grammar learning paradigm with the following specificities: First, we did not use verbal material as verbal processing of grammar may be affected by the patients’ language processing deficits, even in cases where clinical symptoms may indicate otherwise [8]. We implemented a finite state grammar with non-verbal material, notably tones with different pitches [20,26,39]. We used the pitch grammar of Tillmann and Poulin-Charronnat (2010) with controlled ungrammatical sequences that did not differ from grammatical sequences in terms of event frequency, types of bigrams, melodic contour, or anchor tones [20]. Their findings showed that the acquired knowledge went beyond the simple detection of new, previously unheard bigrams, of changes in contour, or of tone repetition. While sequences also differed in terms of associated chunk strength (that is related to familiarity of bi- and trigrams), participants’ data in their task were only influenced by trigram frequency and second-order transitional probabilities but not chunk strength.

Second, we did not use a grammaticality judgment task as results have shown that patients may display task- rather than processing-related deficits [6,40]. This task may entail several cognitive processes, such as memory, decoding ability, and processing speed, which may reduce performance in grammaticality judgments beyond grammar processing [41,42] and may underestimate grammatical knowledge [43]. We developed a paradigm using implicit instructions during exposure and test phases. In the exposure phase, participants were not required to learn or discover grammatical structures, but were asked to detect mistuned tones (occurring in random positions), ensuring attentive listening. In the test phase, we used a cover story: participants were asked to indicate whether each of the test melodies was performed by the same pianist who had played his repertoire in the exposure phase or by another pianist, who had not played before and now presents his own, different repertoire.

Third, we presented the grammatical sequences in a strongly metrical context, which has been shown to lead to processing benefits in perception and learning when compared to irregular metrical contexts or isochronous contexts [26,44,45]. The benefit of a strongly metrical context has been interpreted as facilitated attention in the framework of the Dynamic Attending Theory [46,47]. The Dynamic Attending Theory postulates that stimulus regularities can entrain internal oscillations, which, in turn, guide attention over time and help to develop temporal and perceptual expectations about future events. Listening to strongly metrical patterns leads to the activation of internal oscillations on at least two levels (i.e. a low-oscillatory level and a high oscillatory level, see Method section), and the binding of these oscillations results in the strengthening of temporal expectations (the metric binding hypothesis; [48]). Thus, a strongly metrical context may benefit the learning of a pitch grammar in patients with LIFG lesions (as previously observed in healthy participants, see [26]).

Fourth, as electroencephalography (EEG) is a rather sensitive method to study structure learning [49], we used not only behavioral responses [33,34,50] but also recorded the EEG during exposure and test phases. Indeed, learning might be seen in the EEG data but to a lesser degree in the behavioral data [26,51].

These four methodological changes aimed to ensure the observation of structure learning even under less optimal conditions, such as in brain damaged patients (here with LIFG lesions). In addition, we paid attention to the construction of the test phase aiming to show grammatical structure learning: We compared new grammatical sequences to grammatical sequences containing a subtle ungrammaticality based on a single tone change, rather than on strong violations or random sequences (see Methods). Furthermore, to ensure patients did not suffer from generalized cognitive deficits, we used an oddball task to monitor for selective attention. Finally, patients and their matched controls also performed the Christiansen et al.’s artificial grammar task, which used visual symbols (geometric shapes) and a behavioral grammaticality judgment task (i.e. requiring to explicitly indicate the sequences that followed the same grammatical rules as the ones presented in the exposure phase)^1^. Observing a deficit for learning in this paradigm would extend Christianson et al.’s finding to patients with more circumscribed lesions. Note that as in Christiansen et al., we only recorded behavioral responses for the visual artificial grammar paradigm and not EEG measures. In this experimental material, the visual sequences were presented on the screen by simultaneously showing all items. This presentation format does not allow for time-locking a potential ERP response to the occurrence of an ungrammatical item.

At least two possible outcomes were predicted. First, if the use of non-verbal material, implicit testing, a strong metrical context, and EEG measures make the investigation of structure learning particularly sensitive, we may observe implicit learning of pitch structures in patients despite LIFG lesions. Second, if the LIFG is crucially contributing to artificial grammar learning, no learning should be found in either the auditory or the visual grammar learning conditions. In this case, the results would extend Christiansen et al.’s results from the visual to the auditory modality [34]. Note that it may also be possible to observe above chance performance in the visual artificial grammar condition, but not in the auditory artificial grammar condition. However, this pattern may be observed because of the strong violations used for the ungrammatical items in the test phase for visual items (see procedure of [34]), while relatively subtle violations, which would require grammar knowledge, were used for the pitch material. Consequently, with this result pattern, we would not be able to conclude for a preserved cognitive capacity of implicit learning of grammatical structures.

## Materials and methods

### Participants

**Fig 2:**
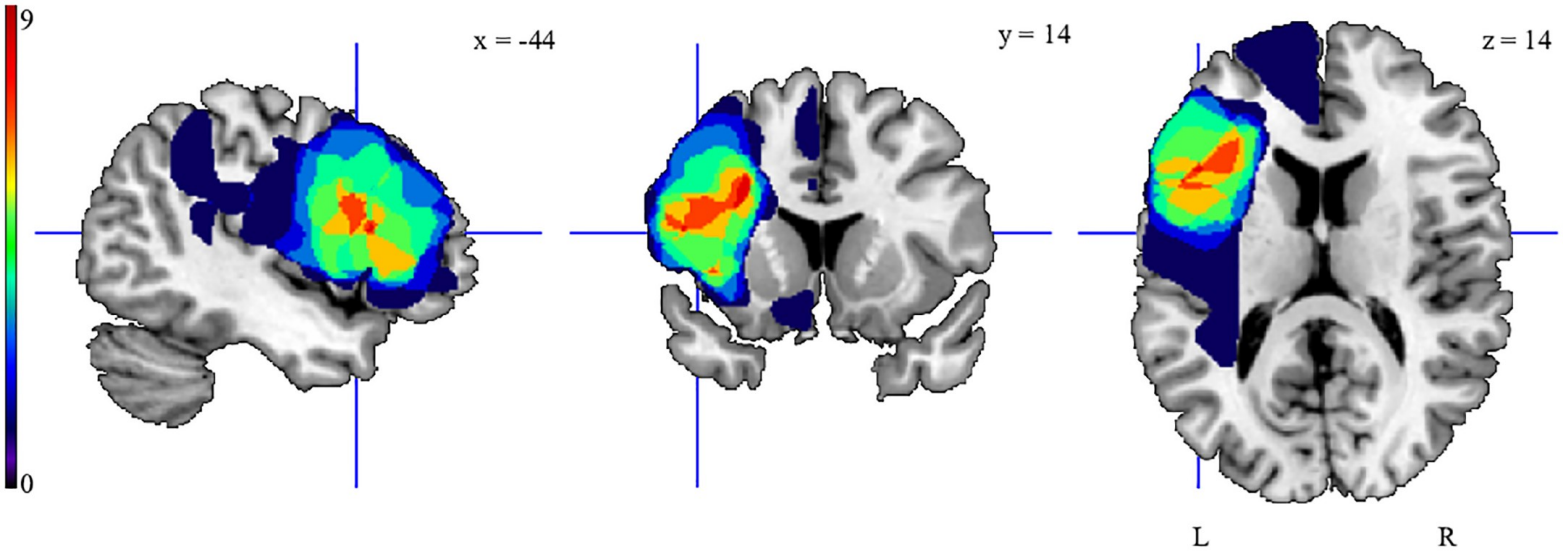
Representation of the lesion distribution of the patients. Colorbar specifies number of patients with overlapping lesions in each voxel, with hot colors indicating a greater number of patients had lesions in the respective region. Maximum lesion overlap was found within the left inferior frontal gyrus. Corresponding Brodmann areas (BA) were identified based on the MNI Brodmann atlas included in MRIcron (https://www.nitrc.org/projects/mricron) as Brodmann area BA 44 (number of overlapping lesions N = 8 at MNI -49, 12, 15) and the underlying subgyral white matter below left BA 44 and 45 (N = 8 at MNI -44, 19, 14 and N = 8 at MNI -28, 14, 30). For this representation, individual T1-weighted images were normalized to Montreal Neurological Institute (MNI) space using the unified segmentation approach as implemented in SPM 12 (Wellcome Department of Imaging Neuroscience, London, http://www.fil.ion.ucl.ac.uk/spm). Lesions were manually delineated by a neurologist (AS) and superimposed on the ch2bet template using the MRIcron software.

We tested nine patients with lesions encompassing the LIFG, involving BA 44 and BA 45, but with intact BG (3 female; mean age of 60.67 years ± 8.54 years, see Table 1 for details on patients’ characteristics). Fig 2 illustrates that the maximum overlap of the patients’ lesions was located in the left inferior frontal gyrus. All patients were initially diagnosed with aphasia, but at the time of testing had only residual aphasic symptoms or no aphasia (see Table 2 for further detailed information about language impairments), while some concomitant cognitive deficits prevailed (Table 2). The diagnosis of persisting (chronic) aphasia at follow-up (performed on average 8.11 months (*SD* = 4.51) after brain injury) was based on the Aachen Aphasia Test (AAT) [52] administered by a trained speech and language pathologist. Aphasia severity was determined by the Stanine-norms for each of the AAT subtests with diagnosis of residual aphasia referring to stanine-scores superior to 5, indicating mild (stanine 5–7) or minimal (stanine 7–9) deficits in all language modalities.

**Table 1:** Presentation of patients’ characteristics.

No	Sex	AatT	H	Education	Tsl	Aetiology	Ls	Lesion location–anatomical reference	Percent damage of BA44	Percent damage of BA45	Lesion volume (ml)	Residual aphasia at follow-up
**P1**	M	64	R	s.e.	3	MCA infarction	L	IFG (op, tri, orb), PrC, INS	20.7	50.2	30.5	Yes
**P2**	F	61	R	h.e.	2	MCA infarction	L	IFG (op, tri), MFG, PrC, OP, INS, CE	56.8	3.4	45.4	Yes
**P3**	M	62	R	l.s.e.	11	MCA infarction	L	IFG (op, tri), PrC, PoC, INS	59.9	0	74.3	Yes
**P4**	M	64	R/L	s.e.	5	MCA infarction	L	IFG (op, tri, orb), MFG, PrC, OP, INS	87.5	95.9	56.4	Yes
**P5**	F	76	R	l.s.e	5	MCA infarction	L	IFG (op, tri), MFG, PrC, INS	22.1	6.4	19.8	No
**P6**	F	60	R	s.e.	5	Postoperative lesion after meningeoma resection	L	IFG (op, tri)	10	3.3	1.3	No
**P7**	M	49	R	s.e.	2	MCA infarction	L	IFG (op, tri, orb), MFG, PrC, OP, INS, IPL, AG, SMG, STG	84.1	85.9	90.0	Yes
**P8**	M	47	R	s.e.	3	SAH with vaso-spastic MCA/ACA infarction	L	IFG (tri, orb), MFG, SFG, SMA, CG, INS	0	76.9	99.7	No
**P9**	M	62	R	s.e.	10	MCA infarction	L	IFG (op, tri, orbi), MFG, PrC, INS	29.4	88.7	32.66	No

Note: The nine control participants (three women) were matched one by one to the patients, they had an average age of 60.22 (SD = 7.64; range 49 to 74), all right-handed, with matched educational levels. Anatomical overlap with relevant brain areas (percent damage of BA44 and 45) was determined using the anatomy toolbox (Version 2.2b) in SPM12 that is based on cytoarchitectonic parcellations of post-mortem brains.

M = male; F = female; R = right; L = left; AatT = Age at testing (years); H = Handedness; Tsl = Time since lesion (years); Ls = Lesion site; h.e. = general certificate of higher education; s.e. = general certificate of secondary education; l.s.e. = general certificate of lower secondary education; MCA = middle cerebral artery; ACA = anterior cerebral artery; SAH = Subarachnoid hemorrhage; IFG = inferior frontal gyrus, op = pars opercularis, tri = triangularis, orb = orbitalis, MFG = middle frontal gyrus, SFG = superior frontal gyrus, CG = cingulate gyrus, PrC = precentral gyrus, PoC = postcentral gyrus, OP = operculum fronto-temporale, INS = insula, IPL = inferior parietal lobule, AG = angular gyrus; STG = superior temporal gyrus, SMG = supramarginal gyrus, CE = cerebellum (CE).

**Table 2:** Summary of patients’ language pathology and cognitive dysfunctions, detailing the presence/absence of residual aphasia at follow-up^1^.

No	Aphasia at follow-up	Language pathology and cognitive dysfunctions
**P1**	Yes	residual non-fluent aphasia with word finding and naming difficulties (initially non-fluent aphasia); associated cognitive impairment with reduced divided attention and working memory performance, increased cognitive interference
**P2**	Yes	residual non-fluent aphasia with word finding difficulties, mild semantic and phonological impairment (initially non-fluent aphasia); reduced working memory capacity and verbal learning^1^
**P3**	Yes	residual non-fluent aphasia with lexical word retrieval deficits, occasionally semantic paraphasias and apraxia of speech (initially global aphasia); associated dysexecutive syndrome^1^
**P4**	Yes	residual amnestic aphasia with lexical and semantic word retrieval deficits (initially non-fluent aphasia); associated attention and verbal working memory deficit, increased cognitive inference^1^
**P5**	No	no aphasia (initially non-fluent aphasia); increased cognitive interference^1^
**P6**	No	non aphasic language symptoms^2^, occasionally word finding difficulties, lexical and semantic word retrieval deficits; associated attention deficit and increased cognitive interference^1^, anxiety disorder
**P7**	Yes	residual non-fluent aphasia, naming and word finding difficulties, agrammatism (initially non-fluent aphasia); reduced working memory capacity and verbal learning^1^
**P8**	No	Non-aphasic language symptoms, occasionally semantic paraphasias and word finding difficulties (initially non-fluent aphasia); reduced working memory capacity and verbal learning, increased cognitive inference^1^
**P9**	No	non-aphasic language symptoms due to impaired cognitive performance (alertness, interference^1^); occasionally word finding/naming difficulties, semantic and formal paraphasias, reduced semantic fluency (initially non-fluent aphasia)

^1^ Neuropsychological diagnoses were based on the Test of Attentional performance (TAP) for attention, the Wechsler Memory Scale Revised (WMS-R) for memory functions, the California Verbal Learning Test (CVLT) for verbal learning, the Color Reading (Stroop) test for cognitive inference, the Behavioural Assessment of the Dysexecutive Syndrome (BADS), and Standardized Link’s Probe (SPL) for executive dysfunctions.

^2^ Patient diagnosed symptomatic epileptic seizures due to left frontal meningeoma (WHO I). Prior to meningeoma resection word finding difficulties and paraphasias were reported by the patient. After resection no more seizures were present and no antiepileptic therapy was initiated. However, a small lesion in the vicinity of the operating area resulted from meningeoma resection. Postoperatively all language deficits alleviated and a mild impairment of lexical and semantic word retrieval and concomitant cognitive deficits were diagnosed. According to the patients’ initial presentation and the presence of a lesion only postoperatively, a gradual cortical reorganization of language functions unlikely occurred prior to operation.

Nine healthy controls were matched for age, gender, handedness, and education to the patient group. None of the participants was wearing hearing aids or reported hearing difficulties. Only for one patient (P5), hearing problems were noticed during the clinical stay, but not further quantified.

The two participant groups did not differ in terms of musical experience (as measured by years of instrumental training, 1.44 years (*SD* = 3.36, ranging from 0 to 10 years) for patients and 2.55 years (*SD* = 4.16, ranging from 0 to 10 years) for controls, *p* = .60). The groups also did not differ in their self-reported sense of rhythm (3.39 (*SD* = 1.24) for the patients and 3.33 (*SD* = 1.12) for controls, *p* = .92) as tested with a subjective scale (from 1 = “I don’t have any sense of rhythm” to 5 = “yes, I have very good sense of rhythm”).

### Materials

#### Auditory artificial grammar learning

The pitch material was based on the artificial grammar of Tillmann and Poulin-Charronnat (2010) [20], which was adapted from a previous grammar [19]. The finite-state grammar contained five tones (a3, a#3, c4, d4, f#4) of a duration of 220 msec and was used to generate sequences for the exposure phase and the test phase (Fig 1). For the exposure phase, 35 grammatical 5-tone and 6- tone sequences were generated (e.g., a#3 c4 c4 d4 c4 and c4 d4 a3 f#4 c4 d4), and two different 5-tone and 6-tone sequences were combined to create sequences of 10 tones and 12 tones [39]. Instead of presenting the tones in an isochronous way (as in [20]), the tone sequences were presented within 14 strongly metrical temporal contexts (see S1 and S2 Sound for examples). These contexts contained inter-onset intervals of 220, 440, 660, 880 msec, respectively. They were constructed to allow the abstraction of a metrical framework, based on oscillatory cycles at two levels (440 and 880 msec). The higher metric level with a period of 880 msec corresponds to the underlying beat of all strongly metrical contexts (see [26] for further information about the metrical temporal structure). In total, 140 different sequences were generated for the exposure phase. To create the mistuned target tones that were used in the exposure phase task, one tone of a grammatical exposure sequence was mistuned by -52 cents (1 semitone = 100 cents). The position of the mistuned tone varied across the sequences from the 2nd to the 9th tone position. Thirty-five exposure sequences contained a mistuned tone and 105 sequences contained only in-tune tones.

For the test phase, 36 other grammatical sequences of either 5 tones or 6 tones were presented within strongly metrical contexts (based on the first halves of the strongly metrical contexts of the exposure phase). Ungrammatical test sequences were created by replacing one grammatical tone in each of the grammatical test sequences by another tone that was part of the finite-state grammar, but that never occurred in this position in grammatical sequences, and thus produced a grammatical violation (e.g., for the grammatical sequence a#3 d4 a3 f#4 a3, the ungrammatical test sequence was a#3 d4 a3 a3 a3; see S3 and S4 Sound for examples). It is important to note that a tone change did not create new bigrams with the preceding and following tones; it only introduced new trigrams of tones (defined as three successive tones). Further, ungrammatical sequences did not differ from grammatical sequences in terms of event frequency, melodic contour, or anchor tones. They differed in terms of bigram- and trigram frequency, associated chunk strength, chunk novelty, and novel chunk position as well as first- and second- order transition probabilities (see [20] for more details). Thirty-six grammatical and 36 ungrammatical test sequences were presented twice during the test phase, resulting in 144 test sequences (72 sequences presented over two test blocks).

#### Visual artificial grammar learning

The visual artificial grammar material was constructed as described in Christiansen et al. (2010) [34]. The finite-state grammar contained five symbols that were used to create visual strings for exposure and test phases. A given string contained 3 to 6 symbols, presented simultaneously and the size of each string was 0.72°. Twenty grammatical strings were used for the exposure phase, and 20 other grammatical strings and 20 ungrammatical strings for the test phase. Ungrammatical test strings were created by replacing one, two, or three symbols in a grammatical string or by removing initial or final elements (thus shortening the strings).

#### Auditory oddball paradigm

Sinusoidal tones of two frequencies were used as standard tones (600 Hz) and deviant tones (660 Hz). The tones had a duration of 50 msec and were presented with inter-onset intervals of 600 msec. In total, 320 standard tones and 80 deviant tones were presented in a pseudo-randomized order via loudspeakers positioned next to the computer screen, respectively. The used standard/deviant ratio was thus 80% vs. 20% (as in [8,53]).

### Procedure

Participants performed the three tasks in a fixed order: First, the auditory oddball paradigm, then the auditory artificial grammar learning task, and then the visual artificial grammar-learning task. The order of the auditory task and the visual task was not counterbalanced because (i) the focus of the current study was on the auditory modality, and the visual paradigm only served as a comparison to Christiansen et al. (2010), and (ii) instructions provided in the visual task (as done by Christiansen et al.) informed participants about the rule-governed grammatical nature of the strings. Consequently, participants may suspect the same features in the auditory modality, and this would render it impossible to have a naïve implicit approach to exposure and test phases. All participants signed informed consent before the experiment. The local ethics committee of the University of Leipzig approved the experimental paradigm and the written informed consent. Participants read a summary of the research protocol and received detailed information about what it means to partake in an EEG experiment. After reading this information, the participants were informed that they could stop the experiment at any point in time. They then had a chance to ask further questions. If this was not the case, they signed a consent form and started the experiment. All participants were capable of following the instructions and signing of the consent form.

In the auditory oddball paradigm, participants were asked to count deviant tones while looking at a white fixation cross on the computer screen in front of them during the EEG recording.

For the main task of the present experiment, the auditory artificial grammar learning paradigm, participants were told that they take part in a music perception experiment without any indication of artificial grammar learning. During the exposure phase, participants were asked to listen carefully to each sequence and to indicate after each sequence whether it contained a mistuned tone or not. The exposure task was explained to participants using three examples with and without mistuned tones. The exposure sequences were presented in random order in two blocks, with one short break between them. No feedback was given after an error. During the test phase, new grammatical and ungrammatical sequences were presented in random order. Participants were asked to indicate which sequence was played by the same pianist who had played his special repertoire in the exposure phase or by another pianist playing another repertoire. The test phase contained two blocks with one short break between them. No feedback was given. In the exposure and test phases, a fixation cross appeared on the screen on average 2000msec (± 500msec) before the presentation of the first tone of each sequence and disappeared with the beginning of the sequence. EEG was recorded during both exposure and test phases.^3^

The visual artificial grammar learning experiment was based on the procedure as described in Christiansen et al. (2010) [34]. We first informed participants that they also take part in a pattern recognition experiment. During the exposure phase, participants were asked to perform a match / mismatch task. On each exposure trial, one grammatical string was presented on the computer screen for 7 sec, followed by a 3-sec delay and then by a second grammatical string presented at the screen for 7 sec. Participants were asked to indicate whether the second string was identical to the first string or not. No feedback was given. In total, 40 pairs of grammatical strings were presented, in which 20 pairs were matched and 20 pairs were mismatched. The exposure phase contained two blocks (with 20 pairs in each block) and a short break between the blocks. After the exposure phase, participants were informed that all strings that were presented in the first part had been generated by a complex set of rules. During the test phase, participants were asked to classify new strings as strings that followed the same rules and as others that did not follow these rules. In total, 40 strings were presented, i.e. 20 grammatical and 20 ungrammatical strings. All symbols of a string were black and were presented on a light grey background on the computer screen. No EEG was recorded.

### Pilot tests

Two pilot tests were run to check that healthy elderly participants can (1) understand and perform the exposure and test phase tasks of the auditory artificial grammar experiment and (2) learn the artificial grammar of visual shapes (as in [34]).

#### Pilot test 1

Eight healthy participants (age range: 55 to 65 years) took part in pilot test 1. The materials and the exposure phase were as described in [26]. The test phase was adapted for the elderly: (1) instead of presenting test sequences by pair, participants responded to each sequence presented separately. (2) we removed the time constraint for responses (participants’ decision making was not limited in time). (3) for the instructions, a cover story presented that two pianists played the melodies: one pianist continues to play the particular repertoire heard in the exposure phase, whereas the second pianist played another repertoire unknown to the participant. The task was to classify the new melodies to melodies played by the same pianist (who played in the exposure phase and thus known to the participant) or played by another pianist. In the exposure phase, correct detection for mistuned tones (hits: 86.43% ± 11.20) and mistuned tone responses for in-tune sequence (false alarms: 20.23%± 9.43) revealed that the elderly participants succeeded the exposure task. In the test phase, performance was above chance level (54.86% (SD = 6.99) correct responses, t(7) = 2.34, p = 0.05). Results thus confirmed that both tasks can be used in the main experiment.

#### Pilot test 2

Six healthy participants (age range: 55 to 65 years) took part in pilot test 2. Material and procedure were as described in [34]. In the exposure phase, percentages of correct responses in the match/mismatch task was 97.08% (±3.69). In the test phase, performance was above chance level (64.58%+ 4.31), *t*(5) = 8.29, *p* < .001). These performance levels were comparable to those of control participants of [34], with 96% (exposure phase) and 63% (test phase).

### Data acquisition and analyses

#### Behavioral data analyses

Data were tested for normality with the Shapiro-Wilk Normality Test. As distributions were not normal for the exposure phases of the auditory and visual artificial grammars and in the oddball task, performance between the participant groups was compared with Mann-Whitney tests for all tasks for the sake of consistency. Note however, that distributions were normal in the test phase. Test phase performance of each participant group was tested against chance level (i.e. 50%) with one-sample t-tests (two-tailed) for auditory and visual artificial grammar learning tasks; performance should be superior to 50% to reflect learning (i.e., correctly categorizing the new items as grammatical or ungrammatical).

#### EEG recording and analyses

Participants were comfortably seated in a sound-attenuated booth in front of a monitor. The EEG signal was recorded from 32 Ag/AgCl electrodes located at standard positions (International 10/20 system sites) via a BrainVision amplifier setup. The sampling rate was 500 Hz. The reference was placed on the left mastoid and the sternum served as ground. The horizontal and vertical electrooculogram (EOG) was recorded. All data were re-referenced offline to averaged mastoids.

Event-related potentials (ERPs) analyses were done with Brain Vision Analyzer software (Brain Products, Munich). Continuous EEG data collected during exposure and test phases were filtered offline with a bandpass filter of 0.1–30 Hz. EEG data containing ocular artifacts were corrected using Independent Component Analysis decomposition by which the components containing a blink or horizontal eye movement were removed [54]. The EEG data were segmented into epochs of 440 msec for grammatical/ungrammatical targets in the test phase, into epochs of 1000 msec for mistune/in-tune targets in the exposure phase and into epochs of 600 msec for standard and deviant tones in the oddball task, all starting with the onset of the target tones or standard/deviant tones and with a 100 msec baseline period before tone onset. Then, we excluded trials from the subsequent analyses based on two criteria, notably trials exceeding 50 μV at the midline electrodes (showing the largest amplitudes) as well as trials with movement artifacts (e.g., facial, auricular muscles) at all other electrodes based on visual inspection. Trials were averaged for each condition and each participant, and then averaged across participants. For the auditory task in the test phase, analyses contained for the grammatical tones on average 59.56 (SD = 11.22) trials for the patients and 51.00 (SD = 8.56) trials for the controls and for the ungrammatical tones on average 60.22 (SD 8.74) trials for the patients and 53.56 (SD = 9.03) trials for the controls. For the exposure phase, the analyses contained for the in-tune tones on average 89.00 (SD = 8.03) trials for the patients and 74.67 (SD = 15.63) trials for the controls and for the out-of-tune tones (maximum = 35) on average 31.33 (SD = 6.30) trials for the patients and 27.44 (SD = 4.45) trials for the controls. For the oddball task, the analyses contained for standard tones on average 155.56 (SD = 62.04) trials for patients and 180.89 (SD = 41.83) trials for controls and for deviant tones on average 56.33 (SD = 11.46) trials for patients and 66.89 (SD = 6.58) trials for controls.

In the test phase of the auditory artificial grammar experiment, ERP mean amplitudes for grammatical and ungrammatical target tones were analyzed in successive 50 msec-time windows from stimulus onset to 400 msec post-stimulus onset. Based on visual inspections and results of statistical analyses in these 50 msec-time windows, a 100–200 msec time window was chosen for the analyses (i.e., the factor item type (grammatical versus ungrammatical) was significant for the windows [100; 150], *p* = .046, and [150; 200], *p* = .043, but not for [200; 250], *p* = .74 and later, *ps* > .22). In the exposure phase of the auditory artificial grammar experiment, mean amplitudes for in-tune and mistuned tones were analyzed in successive 50 msec-time windows from stimulus onset to 1000 msec post-stimulus. Based on visual inspections and results of statistical analyses in these 50 msec-time windows, two latency bands were chosen for the main analyses: 250–400 msec and 550–900 msec. For the earlier windows, the factor item type (in-tune vs mistuned) was significant for the windows [250; 300], *p* = .004, [300; 350], *p* = .003 and [350; 400], *p* = .047, but not for [400; 450], *p* = .39). For the later window, the factor item type emerged with the window [550; 600], *p* = .08, was significant for [600; 650], *p* = .049, marginally significant for [650; 700], *p* = .09; significant for the windows [700; 750], *p* = .01; and [850; 900], *p* = .01; albeit not significant for [750; 850], visual inspection guided us to extend the time window from 550 to 900 msec where the two curves converged.

In the auditory oddball experiment, mean amplitudes for standard and deviant tones were pre-analyzed in successive 50 msec-time windows from stimulus onset to 600 msec post-stimulus. Based on the results of Jakuszeit et al. [8] using time windows of [130–250] and [300–600], visual inspections and results of statistical analyses of differences in amplitude between ERPs at standard and deviant tones in these 50 msec-time windows, two latency windows were chosen for the main analyses: 150–250 msec and 300–550 msec. For the earlier windows, the factor item type (standard vs. deviant) was significant for the windows [150; 200], *p* = .0002, [200; 250], *p* < .0001, but not for [250; 300], *p* = .45). For the later window, the factor item type emerged with the window [300; 350], p = .002, stayed significant for all windows up to 550, all *ps* <001, but was not significant for the window [550; 600], *p* = .91.

A 2x2x2x2 mixed-design ANOVA with item type (two levels, see below), region (anterior vs. posterior), and hemisphere (left vs. right) as within-participant factors and group (patients/controls) as between-participants factor were performed. The factor item type contained the levels grammatical vs. ungrammatical in the test phase, the levels mistuned vs. in-tune in the exposure phase and the levels standard vs. deviant in the oddball task The factors region and hemisphere covered left anterior (F7, F3, FT7, FC3), right anterior (F8, F4, FT8, FC4), left posterior (T7, C3, CP5, P3), and right posterior (T8, C4, CP6, P4) electrode positions.

For the test phase of the auditory artificial grammar experiment (behavioral data and EEG data), a jack-knifing measure was conducted [55] aiming to assure that the result pattern was stable and not dependent on a particular patient inclusion.

Additional analyses on midline electrodes (Fz, Cz and Pz) were performed for the test and exposure phases of the auditory artificial grammar experiment as well as for the oddball task. 2x3 mixed-design ANOVAs with the factors item type (see above for each of the tasks), position (frontal, central, parietal) as within-participant factors and group (patients/controls) as between-participants factor were performed. All p-values reported below were adjusted using the Greenhouse-Geisser correction for non-sphericity, when appropriate, and Tukey tests were used for post-hoc comparisons.

## Results

### Behavioral results

#### Auditory artificial grammar

In the test phase, percentages of correct responses were significantly above chance for the control group (54.40% (*SD* = 5.08); *t*(8) = 2.60, *p* = .032) and just felt short of significance for the patient group (52.62% (*SD* = 3.57); *t*(8) = 2.20, *p* = .059). Performance of the two groups did not differ significantly (*p* = .22; *η*^*2*^ = .08). To further investigate this potential absence of group difference, we performed Bayesian statistics testing for the group effect or its potential absence. While the model supporting Hypothesis 1 showed BF_10_ = .53 (error % = .001) (i.e., with BF inferior to 1 being interpreted as “no evidence”, following the classification of Lee & Wagenmakers, 2014 [56]), the model supporting the null hypothesis showed BF_01_ = 1.89 (error % = .001) (classified as “anecdotal evidence in favor”). The Bayesian analysis (two-tailed) also provided “anecdotal evidence” in favor of performance above chance level for both controls (BF_10_ = 2.58) and patients (BF_10_ = 1.62). The model supporting the null hypothesis showed BF_01_ inferior to 1, suggesting ‘no evidence’ (BF_01_ = .38 and BF_01_ = .62 for controls and patients, respectively).

The jack-knifing measure (Table 3) showed that the performance of the patient and control groups did not differ significantly when excluding one patient and his/her matched control at a time. An additional analysis restricted to the patients with remaining aphasic symptoms (N = 5; and their matched controls; N = 5) confirmed this outcome: percentage of correct responses was above chance for the patient group (54.17% (*SD* = 2.64); *p* = .01) and did not differ from the control group (53.19% (*SD* = 5.95); *p* = .99).

**Table 3:** Results of the jack-knifing approach testing behavioral and EEG data for the test phase of the auditory grammar learning task. Column 1 indicates the patient P and his/her matched control C removed from the presented analysis as well as the result for the entire groups of patients and controls (see main text). The second column indicates the p-values of the Mann-Whitney tests testing for the potential difference between the participant groups in the behavioral task (test phase). The third and fourth columns indicate the p-values of the main effect of item type (grammatical/ungrammatical) and of the interaction between item type and group for the EEG data of the test phase (ROI analysis).

	Behavioral data	EEG data	
	Group difference	Item type effect	Interaction Item type and Group
P1-C1	.23	< 0.01	.16
P2-C2	.05	.01	.27
P3-C3	.32	.02	.47
P4-C4	.14	.03	.18
P5-C5	.16	.02	.61
P6-C6	.53	.03	.43
P7-C7	.53	.03	.46
P8-C8	.53	.05	.28
P9-C9	.29	.03	.11
All participants	.22	.02	.27

In the exposure phase, correct detection for mistuned tones (% of Hits, mean±*SD*) and False Alarms (mistuned tone responses for in-tune sequences, mean±*SD*) were calculated for each participant, and then compared between groups. The two groups differed neither for Hits (68.57 ± 0.18 for patients and 73.02 ± 0.15 for controls, *p* = .*55*, *η*^*2*^ = .02) nor for False Alarms (40.74 ± 0.17 for patients and 31.43 ± 0.13 for controls, *p* = .*30*, *η*^*2*^ = .05*)*. In addition, we calculated the discrimination measure Pr (i.e., [Hits-False Alarms]) that did not differ between patients (0.28±0.23) and controls (0.42±0.20), *p* = .33) and was above chance level (i.e., 0) for both groups (*ps* < .001). These results showed that both groups did the task equally well, suggesting that they were equally attentive during the exposure phase.

#### Visual artificial grammar learning

In the test phase, percentages of correct responses were above chance level for the patient group (59.72% (*SD* = 6.18), *t*(8) = 4.72, *p* < .001) and the control group (61.67% (*SD* = 5.30); *t*(8) = 6.60, *p* < .0001). The two groups did not differ significantly (*p* = .61, *η*^*2*^ = .01). These performance levels were close to the control participants of Christiansen et al. ([34]; 63%), while their patients (N = 7) performed at 51%. Note that when restricting our analysis to the patients with remaining aphasic symptoms, the performance level was similar than to that of the entire group (58.5%; *SD* = 6.75) and above chance level (p = .02).

In the exposure phase, both participant groups performed well in the match/mismatch task (correct responses: 95% (*SD* = 5.3) for patients and 99.44% (*SD* = 1.10) for controls), and their performance did not differ significantly (*p* = .11, *η*^*2*^ = .12).

#### The auditory oddball paradigm

In counting the 80 deviant tones, patients differed from the correct number of deviants by a mean of 5.11 (*SD* = 1.97) and controls differed from the correct number of deviants by a mean of 7.3 (*SD* = 7.91). Performance did not differ between participant groups (*p* = .67, *η*^*2*^ = .008).

### Electrophysiological results

#### Auditory pitch grammar learning

**Fig 3:**
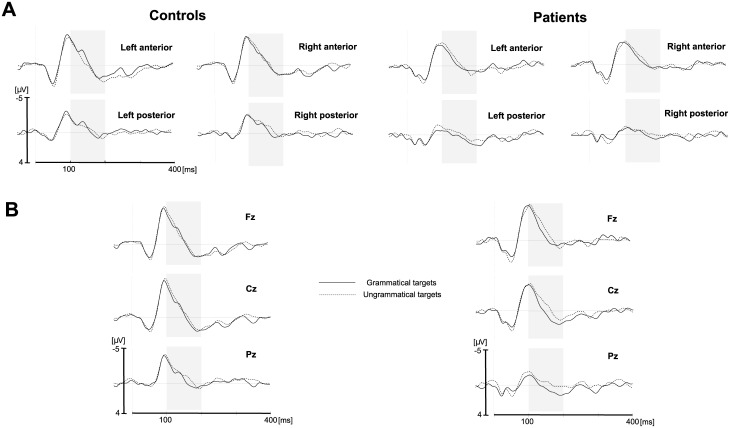
Test phase. A. Grand-average ERPs for grammatical (solid line) and ungrammatical (dashed line) target tones for the control group (left) and the patient group (right). Each line represents the mean of the four electrodes included in the region of interest. B. Grand-average ERPs for grammatical (solid line) and ungrammatical (dashed line) target tones for the control group (left) and the patient group (right) in midline Fz, Cz and Pz electrodes. Light gray areas indicate time windows used for the analyses. (see also Table A in S1 File).

Test phase (see also S1 File for individual data and Table A in S1 File): 1) ROIs: In the 100–200 msec latency window, the main effect of item type was significant: grammatical violations elicited a larger negativity than grammatically correct tones, *F*(1, 16) = 7.45, *p* = .015, partial *η*^*2*^ = 0.32 (grammatical targets, -0.53 μV, ungrammatical targets, -0.87 μV, Fig 3). Item type did not interact with group (*p* = .27). Note that the main effect of item type and the missing interaction between item type and group were confirmed by the jack-knifing measure (Table 3) for each of the patient removals (except for one main effect of item type, which just failed short of significance for the main effect of item type, *p* = .052). Furthermore, the ANOVA showed that the main effect of group was not significant (*p* = .99), and the factor group found expression only in an interaction with region (*F*(1, 16) = 4.65, *p* < .047, partial *η*^*2*^ = 0.23): activation tended to be more negative in anterior regions than in posterior regions for patients (*p* = .07), but not for controls (*p* = .99). Note, however, that the 3-way interaction between group x region and item type was not significant (*p* = .49).

2) Midline analyses confirmed these results: The main effect of item type was significant, *F*(1, 16) = 20.63, *p* < .001, partial *η*^*2*^ = 0.56, with a larger negativity for grammatical violations. This main effect of item type did not interact with group (*p* > .23). In addition, the main effect of position was significant, *F*(2, 32) = 4.85, *p* = .021, partial *η*^*2*^ = 0.23; this was due to the patient group as shown by the interaction between position and group (*F*(2, 32) = 4.65, *p* = .024, partial *η*^*2*^ = 0.23).

As for the behavioral data of the auditory test phase, we further investigated the potential absence of group differences with Bayesian statistics. The model supporting Hypothesis 1 showed BF_10_ = .64 (error % = .003) and BF_10_ = .71 (error % = .004), for ROI and midline analyses respectively (thus with BF inferior to 1 being interpreted as “no evidence”, [56]). The model supporting the null hypothesis showed BF_01_ = 1.57 (error % = .003) and BF_10_ = 1.42 (error % = .004), for ROI and midline analyses respectively, thus being classified as “anecdotal evidence in favor” of no group differences.

**Fig 4:**
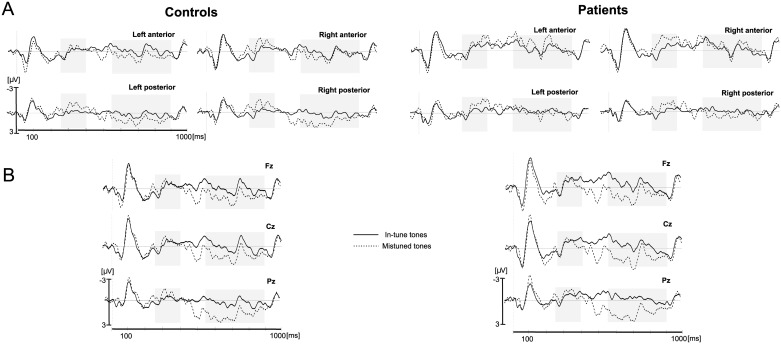
Exposure phase. A. Grand-average ERPs at in-tune (solid line) and mistuned (dashed line) target tones for the control group (left) and the patient group (right). Each line represents the mean of the four electrodes included in each respective region of interest. B. Grand-average ERPs at in-tune (solid line) and mistuned (dashed line) target tones for the control group (left) and the patient group (right) in midline Fz, Cz and Pz electrodes. Light gray areas indicate time windows used for the analyses. (see also Table B in S1 File).

Exposure phase: 1) ROIs: In the 250–400 msec latency window (N2), the main effect of item type was significant: mistuned tones elicited a larger negativity than in-tune tones, *F*(1, 16) = 7.26, *p* = .016, partial *η*^*2*^ = 0.31 (in-tune tones, -0.08 μV, mistuned tones, -0.84 μV, Fig 4, see also Table B in S1 File). No main effect of group was found in this time window nor an interaction with item type (*ps* > .53). In the 550–900 msec latency window (P3), the main effect of item type did not reach significance, *p* = .12 (in-tune tones, -0.04 μV, mistuned tones, 0.38 μV). However, item type interacted with region, *F*(1, 16) = 5.99, *p* = .026, partial *η*^*2*^ = 0.27 (note that the main effect of region was also significant, *F*(1, 16) = 9.63, *p* = .01, partial *η*^*2*^ = 0.38). Post-hoc analyses revealed that the difference between mistuned and in-tune tones was significant only in the posterior region (*p* = .003). The main effect of group was significant (*F*(1, 16) = 10.38, *p* < .005, partial *η*^*2*^ = 0.39), with a larger amplitude for controls than for patients, but no interaction between group and item type was observed (*p* = .16).

2) Midline analyses confirmed these findings. The main effect of item type was significant for the P3, *F*(1, 16) = 4.89, *p* = .042, partial *η*^*2*^ = 0.23, with a significantly larger P3 for mistuned tones, and it was marginally significant for the N2, *p* = .094, with a larger N2 for mistuned tones than for in-tune tones. Most importantly, the interaction between item type and group was neither significant for N2 nor for P3, *ps* > .24. For the P3, a significant interaction between item type and position (*F*(2, 32) = 6.7, *p* = .015, partial *η*^*2*^ = 0.30) suggests a centro-parietal distribution for the difference between mistuned and in-tune tones (*ps* < .01). The main effects of position (*F*(2, 32) = 12.96, p< .0001, partial *η*^*2*^ = 0.45) and group (*F*(1, 16) = 7.86, p = .01, partial *η*^*2*^ = 0.33) were also significant.

#### Auditory oddball paradigm

**Fig 5:**
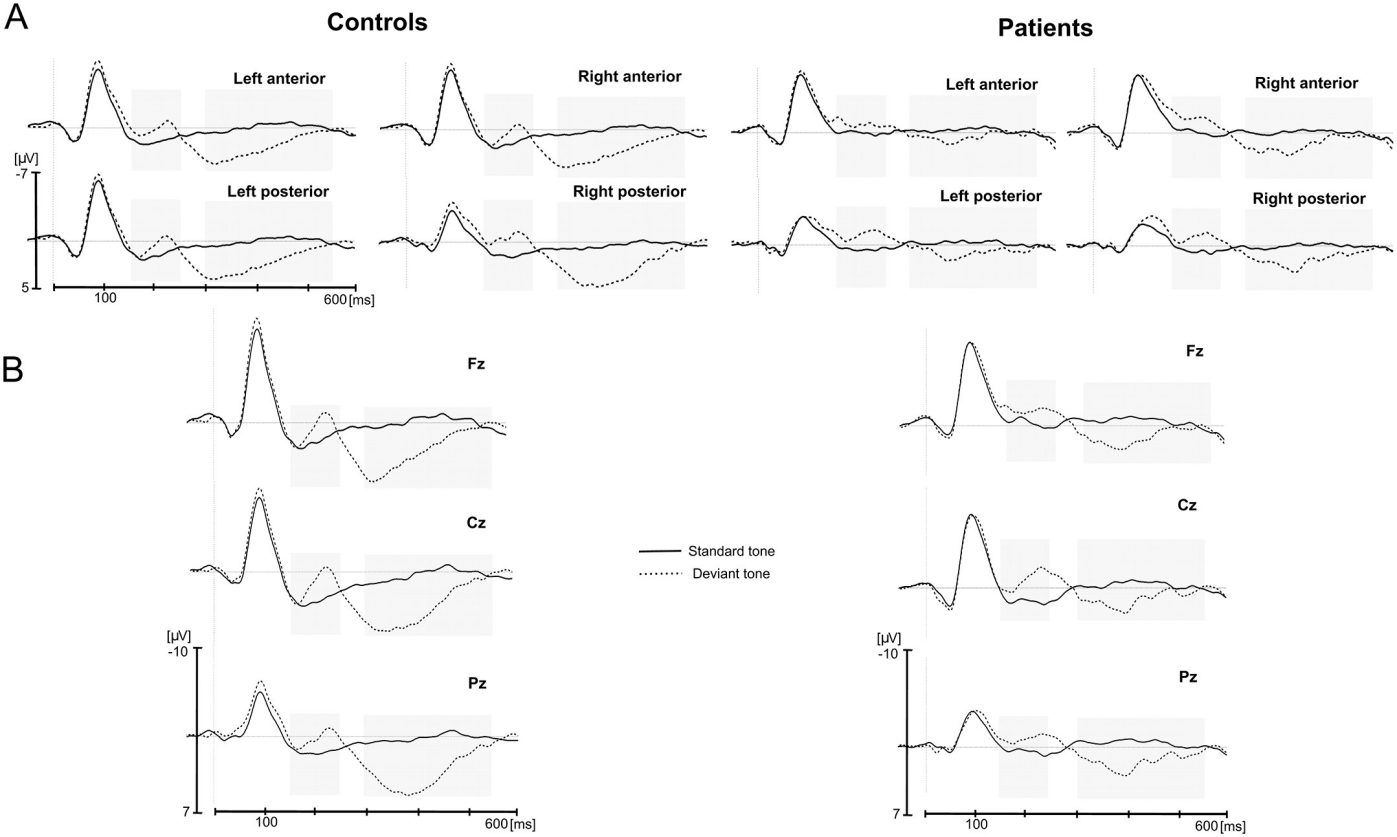
Oddball auditory task. A. Grand-average ERPs at standard (solid line) and deviant (dashed line) tones for the control group (left) and the patient group (right). Each line represents the mean of the four electrodes included in each respective region of interest. B. Grand-average ERPs at standard (solid line) and deviant (dashed line) tones for the control group (left) and the patient group (right) in midline Fz, Cz and Pz electrodes. Light gray areas indicate time windows used for the analyses. (see also Table B in S1 File) Note that standard tones were presented with a probability of .8 and deviant tones with a probability of .2.

1) ROIs: In the 150–250 ms latency window (N2), the main effect of item type was significant, *F*(1, 16) = 31.48, *p* < .0001, partial *η*^*2*^ = 0.66: deviant tones elicited a larger negativity than did the standard tones (standard tones, 0.68 μV, deviant tones, -0.66 μV, Fig 5). The interaction between item type and group was not significant, *p* = .89, nor was the main effect of group (*p* > .25).

In the 300–550 ms latency window (P3), the main effect of item type was significant, *F*(1, 16) = 33.58, *p* < .0001, partial *η*^*2*^ = 0.68: the deviant tones elicited a larger positivity than did the standard tones (standard tones, -0.15 μV, deviant tones, 1.58 μV). The interaction between item type and group was significant and showed that the deviant tones elicited a larger amplitude of the P3 in the control group than in the patient group (*F*(1, 16) = 5.00, *p* = .04, partial *η*^*2*^ = 0.24). Post-hoc analyses revealed that the P3 difference between deviant and standard tones was significant for the control group (*p* < .001), but only marginally significant for the patient group (*p* = .095; -0.38 μV for standard tones and 0.68 μV for deviant tones, Fig 3, see also Table B in S1 File). Furthermore, item type interacted significantly with hemisphere (*F*(1, 16) = 11.49, *p* < .004, partial *η*^*2*^ = 0.42) (note that the main effect of hemisphere was significant too; *F*(1, 16) = 15.73, *p* < .001, partial *η*^*2*^ = 0.50): the amplitude evoked by the deviant tones was larger in the right than left hemisphere (*p* < .0002), while this was not significant for the standard tones (*p* = .39).

2) Midline analyses confirmed the main effect of item type for N2 and P3: deviant tones elicited a larger N2, *F*(1, 16) = 17.13, *p* < .001, partial *η*^*2*^ = 0.52, and a larger P3, *F*(1, 16) = 30.66, *p* < .0001, partial *η*^*2*^ = 0.66. For the N2, the interaction between item type and group as well as the main effect of group were not significant, *p* = .65 and *p* = .18, respectively. For the P3, the interaction between item type and group just fell short of significance, *p* = .07, with a stronger item type effect for the control group (p < .001) than for the patient group (*p* = .09). Note that the main effect of group was significant, *F*(1, 16) = 4.92, *p* = .04, partial *η*^*2*^ = 0.24, as was the interaction between item type and position, *F*(2, 32) = 3.75, *p* = .04, partial *η*^*2*^ = 0.19. In addition, for the N2, the main effect of position was significant, *F*(2, 32) = 4.36, *p* < .05, partial *η*^*2*^ = 0.21, with its maximum at Cz.

## Discussion

The aim of the present study was to investigate whether patients with lesions encompassing the LIFG can learn new grammatical pitch structures. Aiming to maximize learning and test sensitivity, we chose implicit exposure and test phases, non-verbal material, regular temporal presentations (i.e. strongly metrical presentation), and the use of EEG to test patients. In addition, we used an auditory oddball task to test for potential deficits in selective attention. Behavioral results as well as the N2 response to deviant tones in the oddball task showed comparable results between groups, while differences in P3 amplitude size were evident in patients compared to controls. These findings suggest somewhat spared, albeit potentially altered attentional processes as reflected in these two components for the patient group. More specifically, the comparable N2 response in patients and controls indicates that patients can voluntarily detect a deviant tone in a sound sequence when attention is directed to detecting deviant sound properties (e.g. [57]). On the other hand, a reduction of the P3 amplitude in response to deviant tones in the patients may indicate that they are less capable than controls to adapt their mental representation of the expected sound quality (e.g. [58,59]). Consequently, the current results show that, despite the reduced P3 in the oddball task, patients could attentively listen to and detect changes in tone sequences as well as learn the artificial grammar, as suggested by the results of the exposure and test phase.

The behavioral results of the test phase showed that control participants learned the artificial grammar, as suggested by above chance level performance. While patients’ performance was only marginally significantly above chance level, their performance did not differ from controls’ performance, suggesting that also patients became at least somewhat sensitive to the rather subtle grammatical violations. Congruently, the ERPs showed an enhanced negativity in response to ungrammatical targets (in comparison to grammatical targets) in both participant groups. Implicit measures (i.e., participants were never told about the underlying grammar) used in the test phase may be more beneficial when evaluating implicit learning than grammaticality judgments often used in seminal artificial grammar studies (e.g., [60]). The capacity to learn artificial non-verbal grammars independently of modality was corroborated by the results of the visual grammar-learning task based on shapes (but see below). According to these results, it stands to reason that the LIFG does not play an exclusive role in non-verbal artificial grammar learning and may be part of a larger neural network supporting implicit learning.

The fact that we observed implicit learning in LIFG lesion patients is surprising in light of previous neuroimaging results that reported LIFG activation during artificial language learning in the exposure phase [61], and artificial grammar learning in the test phase [4,30,32,62]. Furthermore, while the right IFG (RIFG) was also activated in some of these fMRI studies [14,30,32], additional data by Flöel et al. (2009) suggest the predominance of the LIFG in artificial grammar learning [63]. Using diffusion tensor imaging, Flöel et al. tested whether white matter integrity of fibers arising from Broca’s area was related to the acquisition of an artificial grammar based on letters. Results showed that inter-individual variability in the performance of young adults correlated with the white matter integrity in fibers originating in the LIFG, but not with its right-hemispheric homologue (RIFG). Antonenko et al. (2012) further found that grammaticality judgment in older adults was positively correlated with fractional anisotropy of white matter microstructure underlying LIFG and RIFG and with fractional anisotropy of the tracts originating in the LIFG only [31]. These studies [30–32,63] all used visual materials. It may be argued that the left-hemisphere dominance is anchored in the verbal nature of the material (in particular as [64] reported right-hemisphere dominance for the statistical learning of visual shapes). Similarly, in the current case, it may be argued that the processing of pitch is driven by right hemisphere correlates, notably the RIFG, as previously observed for musical syntax processing [3,65,66]. Note, however, that some of these studies on musical syntax processing also reported bilateral IFG activation, even though the LIFG was activated to a lesser extent. Importantly, Sammler, Koelsch, and Friederici (2011) reported that patients with lesions in Broca’s area show deficits in musical structure processing, suggesting a rather domain-general function of the LIFG [67]. Even though we cannot exclude the possibility that an intact RIFG may have facilitated the learning of an artificial pitch grammar, the present data show that the LIFG seems not to be necessary for the implicit learning of new non-verbal grammars despite its previously attributed role in implicit learning and structure processing.

Along similar lines of reasoning, it may be argued that intact artificial grammar learning in LIFG patients may be observed because either the RIFG compensates LIFG dysfunction or the LIFG is part of an integrated (probably bilateral) neural network supporting grammar learning. Indeed, beyond the RIFG, the BG have been implicated in sequence learning and syntax processing [38,68,69]. It is known that the BG project to Broca’s area [70] and that both structures are relevant for procedural memory-related processes [71,72]. An IFG/BG interface has also been discussed with regards to temporal processing [38,73,74]. Both the IFG and the BG—among other areas (cerebellum, thalamus, and cortical structures)—are part of a temporal processing network [45]. With regards to the current stimulus set, the strongly metrical context, in which the artificial pitch grammar was embedded in, may thus have supported the artificial grammar acquisition. Based on previous findings [32,75], one might further wonder whether the undamaged temporal lobe might have also contributed to the patients’ test phase data, at least for the part of the ungrammatical changes related to associated chunk strength (e.g., [76]) or string familiarity (even though these changes did not contribute to previous learning data in healthy participants in [20]). It would thus be interesting in a future study to manipulate string familiarity versus other structural features (including perceived item similarity, e.g., [77]) to further determine the potential contribution of intact temporal lobe structures versus the deficit due to the LIFG lesions (see [23] for further discussion).

A recent study using an artificial language provides further insight into our data. Goranskaya et al. (2016) suggest that the LIFG, which has been shown to be more strongly activated for complex structures than for simple structure processing as well as for the processing of structure violations than intact structures, might not contribute to successful artificial grammar learning, but might be rather involved in rule application and representation during the test phase [78]. This suggest that our patient group could learn during the exposure phase despite their lesions, and in the test phase, the implicit paradigm implementation and the EEG measurements allowed revealing their detection of ungrammatical features in the newly learned experimental material.

As summarized above, the present results show that LIFG patients are capable of amodal (auditory, visual) artificial grammar learning. Independent of modality, participants perceived differences between grammatical and ungrammatical structures in the test phase and thus showed implicit learning of an artificial grammar. The visual grammar learning task was a replication of Christiansen et al. (2010) who had reported impaired artificial grammar learning in agrammatic aphasics [34]. This difference of results may be due to patients having more varied and extended lesions in Christiansen et al.’s study (2010), leading to more severe symptoms than in the current patient sample. For example, aphasics with extended fronto-striatal lesions often display more severe aphasic symptoms [36,37]. As the IFG and the BG both contribute to implicit learning [32,79], lesions in both areas may be essential to result in impaired artificial grammar learning. Thus, one possible future direction of the current results would be to investigate artificial grammar learning of pitch structures and of visual shapes in patients with focal BG lesions.

However, caution is needed to interpret the results of the visual material. The ungrammaticalities used in Christiansen et al. (2010) introduced relatively strong structure violations (one to three elements changed in each string or an initial or final element was removed). We can thus speculate that the performance level in the visual task may reflect these strong local violations in the ungrammatical strings, and thus the data do not allow concluding for unimpaired structure learning in the visual modality (see [21,23,80], for a similar rational). In contrast, the ungrammatical *auditory* sequences contained rather subtle violations, that is, only one tone in the grammatical test sequence was changed for one other tone (that was part of the grammar), and this change did not create new bigrams with preceding and following tones. The results revealed that patients can detect subtle grammatical violations in an auditory pitch grammar. This was also reflected in the ERP results, showing a larger early negativity in response to ungrammatical target tones compared to grammatical tones for patients and controls. Future research now needs to implement our approach of implicit learning and implicit testing (including the use of fine violations in the testing material) in patients with circumscribed IFG lesions in the visual modality, with non-verbal material (as in [34]), but also with verbal material (in either auditory or visual modalities). A first attempt of comparing learning of verbal and tonal structures was recently done with vascular and progressive non-fluent aphasic patients [81]. While both patient groups performed below the control group, they also showed learning. However, this may also include the detection of local violations (such of new, not previously encountered bigrams), similarly as for Christiansen et al. (2010) [34].

Regarding the current study, we suggest that the strongly metrical presentation may have facilitated implicit learning of the pitch grammar in patients and matched controls. Two previous studies with young healthy participants reported that a strongly metrical context boosts artificial grammar learning of pitch structures in comparison to a temporally irregular context [39] and compared to an isochronous context [26]. Here we used a strongly metrical context to help patients to process tones in the to-be-learned structure. In line with the Dynamic Attending Theory [47,48], we suggest that a strongly metrical context facilitates the synchronization of to-be-processed events with internal neural oscillations, which guide attention over time and allow developing temporal expectations about future tones. The presentation of an artificial pitch grammar in a strongly metrical context may therefore engage temporal processing network(s) [38,45], which allow detecting temporal regularities in the sensory input and to predict future events in order to optimize cognitive and behavioral performance. This subcortico-cortical temporal processing network aims (i) at the extraction of temporal regularities of external events, for example, in speech or music, and (ii) at the generation of temporal expectations that facilitate auditory processing. In Selchenkova et al. [26], grammatically incorrect target tones elicited a larger negativity than grammatically correct target tones in a similar time window as observed here (150–350 ms). In line with our previous results [26,39], we suggest that a strongly metrical context allows perceivers to develop temporal expectations about future events and thus facilitate the learning of an artificial pitch grammar.

## Conclusion

The present study investigated artificial pitch grammar learning in patients with well-described LIFG lesion sites. We observed that LIFG patients were able to learn a pitch grammar embedded in a strongly metrical context. They also learned an artificial grammar of visual shapes. These results suggest that the LIFG is part of a neural network engaged in artificial grammar learning, but does not play an exclusive role and may be compensated by other areas within this network when function of the LIFG is disrupted. In the present study, we aimed at maximizing learning and test sensitivity by using, among others, implicit exposure and test phases. Observing learning in the present patient sample is encouraging the use of implicit approaches also in other patient groups (e.g.,[82]) before concluding that cognitive capacity is restricted by a lesion. Our results also motivate three further research directions, in particular (1) to investigate the potentially causal interpretation of our findings with the role of the LIFG in non-verbal (pitch) structure learning, such as for example by using brain stimulation techniques (as for verbal structure learning in [5]), (2) to manipulate the used grammatical violations to further study the involved brain structures (distinguishing the involvement of different frontal areas as well as temporal areas, e.g., [75]) and potential dynamic interactions between brain structures in the neural network underlying grammar learning as well as (3) to further investigate the developing brain for artificial structure learning, notably by extending previous research on first language learning, which studies the maturation of cerebral networks (including left frontal cortex and temporal cortex) in the development of syntax acquisition (e.g., [83,84]).

## Supporting information

S1 Sound An example item of the 10-tone exposure sequences. (MP3)

S2 Sound An example item of the 12-tone exposure sequences. (MP3)

S3 Sound An example item of the grammatical test sequences (here with 5 tones). (MP3)

S4 Sound An example item of the ungrammatical test sequences, matched with the grammatical test sequence of S3 Sound (note: The same type of construction applies for the 6-tone test sequences). (MP3)

S1 File Supplementary material. (DOCX)
