# Supplementary material for: Implicit learning of artificial grammatical structures after inferior frontal cortex lesions
Source: PLoS One. 2019 Sep 20;14(9):e0222385. doi: 10.1371/journal.pone.0222385 (PMC6754135; doi:10.1371/journal.pone.0222385)
Supplement: S1 File — (DOCX) [file pone.0222385.s005.docx]

***Supplementary Material***

*Auditory pitch grammar learning – Test phase:*

The figures below plot the individual data for the difference between ERP amplitudes for grammatical and ungrammatical targets for patients and controls for the midline electrodes (top) and the ROI analyses (middle) as well as the individual behavioral data with the accuracy for patients and controls (bottom).


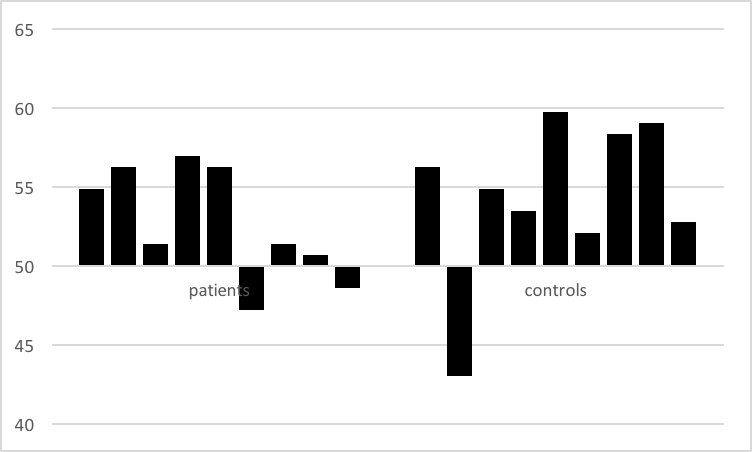


Table A. Test Phase: Numerical difference (in μV) between ERP amplitudes for ungrammatical and grammatical target tones in 100-200ms time window in the regions of interest and midline electrodes for the control and patient groups. Negative values indicate that the response was more negative for ungrammatical targets in comparison to grammatical targets.

| Region of interest or midline electrode | Controls | Patients |
| --- | --- | --- |
| Left anterior | 0.09 | -0.59 |
| Right anterior | -0.23 | -0.38 |
| Left posterior | -0.18 | -0.60 |
| Right posterior | -0.48 | -0.39 |
| Fz | -0.35 | -0.71 |
| Cz | -0.56 | -1.00 |
| Pz | -0.58 | -0.93 |

Table B. Numerical difference (in μV) between ERP amplitudes in the regions of interest and midline electrodes for the control and the patient groups. Oddball task: Difference between ERPs for deviant and standard tones in 150-250 ms (first column; negative values indicate more negative amplitude for deviant tones) and 300-550 ms (second column, positive values indicate more positive amplitude for deviant tones) time windows. Exposure phase: Difference between ERPs for mistuned and in-tune target tones in 250-400 ms (first column, negative values indicate more negative amplitude for mistuned tones) and 550-900 ms (second column; positive values indicate more positive amplitude for mistuned tones) time windows.

|  | Oddball task | | | | Exposure phase | | | |
| --- | --- | --- | --- | --- | --- | --- | --- | --- |
| Region of interest or midline electrode | Controls | | Patients | | Controls | | Patients | |
| Left anterior | -1.18 | 2.08 | -0.71 | 0.60 | -0.18 | 0.86 | -0.66 | -0.67 |
| Right anterior | -0.83 | 2.34 | -1.67 | 1.20 | -0.91 | 0.38 | -1.08 | -0.03 |
| Left posterior | -1.85 | 2.42 | -1.35 | 0.91 | -0.76 | 0.85 | -0.81 | 0.21 |
| Right posterior | -1.61 | 2.72 | -1.46 | 1.53 | -0.98 | 1.08 | -0.74 | 0.67 |
| Fz | -0.97 | 2.74 | -1.20 | 1.60 | -0.26 | 1.09 | -0.85 | -0.17 |
| Cz | -1.54 | 3.47 | -2.31 | 1.13 | -0.22 | 1.28 | -0.81 | 0.32 |
| Pz | -1.34 | 3.81 | -1.33 | 2.04 | -0.56 | 1.81 | -0.89 | 1.04 |
